# α‐galactosylceramide generates lung regulatory T cells through the activated natural killer T cells in mice

**DOI:** 10.1111/jcmm.14008

**Published:** 2018-11-13

**Authors:** Qianhui Chen, Xuxue Guo, Nishan Deng, Linlin Liu, Shuo Chen, Ailing Wang, Ruiyun Li, Yi Huang, Xuhong Ding, Hongying Yu, Suping Hu, Hanxiang Nie

**Affiliations:** ^1^ Department of Respiratory Medicine Renmin Hospital of Wuhan University Wuhan China; ^2^ Nursing Department Wuhan University School of Health Sciences Wuhan China

**Keywords:** α‐galactosylceramide, interleukin ‐2, invariant natural killer cells, regulatory T cells

## Abstract

Our previous study showed that intraperitoneal injection of α‐galactosylceramide (α‐GalCer) has the ability to activate lung iNKT cells, but α‐GalCer‐activated iNKT cells do not result in airway inflammation in wild‐type (WT) mice. Many studies showed that iNKT cells had the capacity to induce Treg cells, which gave rise to peripheral tolerance. Therefore, we examined the influence of intraperitoneal administration of α‐GalCer on the expansion and suppressive activity of lung Treg cells using iNKT cell‐knockout mice and co‐culture experiments in vitro. We also compared airway inflammation and airway hyperresponsiveness (AHR) after α‐GalCer administration in specific anti‐CD25 mAb‐treated mice. Our data showed that intraperitoneal injection of α‐GalCer could promote the expansion of lung Treg cells in WT mice, but not in iNKT cell‐knockout mice. However, α‐GalCer administration could not boost suppressive activity of Treg cells in WT mice and iNKT cell‐knockout mice. Interestingly, functional inactivation of Treg cells could induce airway inflammation and AHR in WT mice treated with α‐GalCer. Furthermore, α‐GalCer administration could enhance iNKT cells to secrete IL‐2, and neutralization of IL‐2 reduced the expansion of Treg cells in vivo and in vitro. Thus, intraperitoneal administration of α‐GalCer can induce the generation of lung Treg cells in mice through the release of IL‐2 by the activated iNKT cells.

## 1 INTRODUCTION

Invariant natural killer T (iNKT) cells belong to a noval and relatively rare subpopulation of T lymphocytes that are characterized by a semi‐invariant T‐cell receptor α‐chain (Vα14‐Jα18 in mice and Vα24‐Jα18 in humans) and NK cell markers, NK1.1.^1^, ^2^ Invariant natural killer T cells have the capacity to recognize glycolipid antigens, presented by the non‐polymorphic major histocompatibility complex class I‐related molecule CD1d.^2^, ^3^ It has been identified that iNKT cells recognize α‐galactosylceramide (α‐GalCer), a potent and specific glycolipid originally derived from marine sponges.^4^ When activated, iNKT cells are able to promptly secrete copious amounts of cytokines, including Th1‐type cytokines like interferon (IFN)‐γ and Th2‐type cytokines like interleukin (IL)‐4, IL‐5 and IL‐13, within hours.^5^, ^6^ Through this ability, the activated iNKT cells can regulate various immune pathways, thus, are considered to be an immunomodulatory T‐cell subset, bridging innate and adaptive immune responses.^5^, ^6^, ^7^ Recently, iNKT cells activated by α‐GalCer were shown to promote the expansion and suppressive activity of regulatory T (Treg) cells to prevent myasthenia gravis in mice.^8^ Ronet et al.^9^ reported that the activation of iNKT cells by the administration of α‐GalCer prior to *Toxoplasma gondii* infection can augment the frequency of IL‐10‐secreting Treg cells to reduce inflammation in ileitis. These findings highlight that iNKT cells have the ability to induce Treg cells, which result in peripheral tolerance. However, much less is known whether α‐GalCer can induce the generation of lung Treg cells through the activation of iNKT cells to promote airway tolerance.

Airway exposure to potential environment allergens can lead to immunological tolerance, and Treg cells play a crucial role in the development of the airway homeostatic state and limiting airway inflammation related to allergic asthma.^10^, ^11^ In our previous study, we found that intraperitoneal administration of α‐GalCer had the ability to stimulate iNKT cells, but α‐GalCer‐activated iNKT cells do not elicit airway inflammation in wild‐type (WT) mice in the absence of ovalbumin (OVA) immunization and challenge.^12^ At present, it is proposed that iNKT cells have the capacity to induce Treg cells, which give rise to peripheral tolerance.^8^, ^9^ Thus, it was hypothesized that intraperitoneal administration of α‐GalCer may induce the generation of lung Treg cells through the activation of iNKT cells in naive mice.

To verify this hypothesis, we have investigated the expansion and suppressive activity of lung Treg cells using iNKT cell‐knockout mice and co‐culture experiments in vitro. We also compared airway inflammation and airway hyperresponsiveness (AHR) after α‐GalCer administration in specific anti‐CD25 mAb‐treated mice. Our data demonstrate that intraperitoneal administration of α‐GalCer can induce the generation of lung Treg cells in mice through the release of IL‐2 by the activated iNKT cells.

## 2 MATERIALS AND METHODS

### 2.1 Mice

Wild‐type BALB/c mice, 6‐8 week old, were purchased from the Center of Animal Experiment of Wuhan University (Wuhan, China). CD1d‐knockout mice on BALB/c background were obtained from The Jackson Laboratory (Bar Harbor, ME). All mice were female and maintained under environmentally controlled and specific pathogen‐free conditions (22°C, 12 hours light/12 hours dark cycle) at the animal Biosafety Level three Laboratory of the Center of Animal Experiment of Wuhan University (Wuhan, China). All animal care and handling procedures were in accordance with the Institutional Ethics Committee of Wuhan University.

### 2.2 In vivo administration of α‐GalCer

A stock solution of α‐GalCer (KNR7000) (Enzo Life Sciences, Ann Arbor, MI) was diluted into 0.01 mg/mL in 0.5% polysorbate‐20 and stored at −20°C for further study. The intraperitoneal injection was used as the route of administration of α‐GalCer, as previously reported.^13^ In some experiments, intravenous administration of α‐GalCer was served as control. Mice were intraperitoneally administrated or intravenously injected via tail vein with 2 μg of α‐GalCer. Control mice were intraperitoneally injected with the same amount of 0.5% polysorbate‐20 in PBS alone.

### 2.3 Airway tolerance and Th2 inflammatory responses

The protocol was performed according to the report as previously described.^14^ Briefly, BALB/c mice were intraperitoneally injected with 2 μg of α‐GalCer in 0.5% polysorbate‐20 or the same volume of 0.5% polysorbate‐20 in PBS. After 9 days, mice were immunized by intraperitoneal injection with 50 μg of chicken OVA (grade V; Sigma, St. Louis, MO) adsorbed to 2 mg of aluminium hydroxide (Thermo Scientific Pierce, Rockford, IL). Another 9 days later, mice were challenged with intranasal administration of 50 μg of OVA in PBS on days 18, 19 and 20. Airway hyperresponsiveness was measured 24 hours after the final challenge, and then bronchoalveolar lavage fluid (BALF) and lungs were obtained for further analysis.

### 2.4 In vivo Ab administration

For selective depletion of CD25^+^ T cells, 500 μg of anti‐CD25 mAb (clone PC61; BD Pharmingen, San Diego, CA) or IgG isotype mAb was intravenously administrated into mice. A total of 150 μg of anti‐IL‐2 mAb (IgG2a, clone S4B6; BD Pharmingen) or IgG isotype mAb was intravenously administrated into mice for initial neutralization of IL‐2. After resting for 72 hours, the mice were intraperitoneally injected with α‐GalCer or PBS. Three days later, mice were killed for further study.

### 2.5 Quantitative RT‐PCR for FoxP3 and IL‐2

To determine mRNA expression, total RNA was extracted using TRIzol (Invitrogen, CA), and cDNAs were synthesized with a Revertaid first‐strand cDNA synthesis kit (ShineGene, Shanghai, China). Quantitative real‐time (RT) PCR was accomplished in triplicate with SYBR Green Supermix (ShineGene, Shanghai, China) following the manufacturer's instructions. Primers used in PCR are as follows: For FoxP3, forward, 5′‐CCAGATGTTGTGGGTGAGTG‐3′ and reverse, 5′‐AGAGCCCTCACAACCAGCTA‐3′, for IL‐2, forward, 5′‐TGAACTTGGACCTCTGCG‐3′ and reverse, 5′‐ATTGAGGGCTTGTTGAGA‐3′, and GAPDH, forward, 5′‐ATGGGTGTGAACCACGAGA‐3′ and reverse 5′‐CAGGGATGATGTTCTGGGCA‐3′. The data were normalized on the basis of the expression levels of GAPDH, and the interest gene expression was analysed using 2^−▵▵Ct^.

### 2.6 Splenocytes culture in vitro

Spleen mononuclear cells (MNCs) (5 × 10^6^ cells/well) from WT mice or CD1d‐knockout mice intraperitoneally treated with α‐GalCer or PBS were cultured together with α‐GalCer (100 ng/mL) in round‐bottom 96‐well plates pre‐bound with anti‐CD3 (2 μg/mL) and anti‐CD28 (2 μg/mL) (all from Becton Dickinson, Franklin Lakes, NJ) in RPMI 1640 complete medium (Invitrogen, CA). Three days later, supernatants were harvested to determine the concentration of IL‐2 by ELISA according to the manufacturer's instructions (eBioscience, San Diego, CA). Cellular components were obtained for RNA extraction and the expression of IL‐2 mRNA was determined by quantitative RT‐PCR.

### 2.7 Isolation of CD4^+^ T cells, CD4^+^ CD25^−^ T cells and CD4^+^ CD25^+^ T cells

CD4^+^ CD25^+^ T cells were isolated from spleens of mice 72 hours after intraperitoneal or intravenous treatment of α‐GalCer or PBS, whereas CD4^+^ T cells and CD4^+^ CD25^−^ T cells were isolated from spleens of WT mice. For the isolation of CD4^+^ T cells, non‐CD4^+^ T cells were first magnetically labelled and retained in the magnetic activated cell sorting (MACS) columns (Miltenyi Biotec, Auburn, CA), and CD4^+^ T cells were eluted from the column for enrichment. For obtaining CD4^+^ CD25^−^ T cells and CD4^+^ CD25^+^ T cells, the enriched CD4^+^ T cells were labelled, in parallel, with anti‐CD25‐PE. Subsequently, the CD4^+^ T cell fraction was magnetically labelled again with anti‐PE magnetic microbeads and loaded onto a MACS column placed in the magnetic field of a MACS Separator (Miltenyi Biotec) following the manufacturer's procedures. CD4^+^ CD25^+^ T cells and CD4^+^ CD25^−^ T cells were harvested for further study.

### 2.8 CD4^+^ CD25^+^ T cell culture in vitro

CD4^+^ CD25^+^ T cells (1 × 10^5^ cells/well), defined as Treg cells,^15^ from mice intraperitoneally or intravenously treated with α‐GalCer or PBS in round‐bottom 96‐well plates pre‐bound with anti‐CD3 (2 μg/mL) and anti‐CD28 (2 μg/mL) in RPMI 1640 complete medium. Three days later, supernatants were harvested to determine the concentration of IL‐10 by ELISA according to the manufacturer's instructions (eBioscience).

### 2.9 Assay of suppressive activity of CD4^+^ CD25^+^ T cells

CD4^+^ CD25^−^ T cells (1 × 10^5^ cells/well) were used as responder T cells and co‐cultured with CD4^+^ CD25^+^ T cells at the indicated ratio (0:1; 0.5:1; 1:1) of Treg/CD4^+^ CD25^−^ T in round‐bottom 96‐well plates pre‐bound with anti‐CD3 (2 μg/mL) and anti‐CD28 (2 μg/mL) in RPMI 1640 complete medium. Three days later, supernatants were harvested to determine the levels of IL‐4 and IFN‐γ by ELISA according to the manufacturer's instructions (eBioscience).

### 2.10 Invariant natural killer T cell isolation and co‐culture with CD4^+^ CD25^−^ T cells

To isolate lung iNKT cells, the lungs from WT mice intraperitoneally treated with α‐GalCer were removed, cut into small pieces, digested with collagenase I (1 mg/mL, Invitrogen, CA) at 37°C for 1 hour and filtered via a 100‐μm nylon net filter. The lung MNCs were sorted by centrifugation at 800 ×  *g* for 20 minutes at room temperature in a Lymphoprep gradient (density = 1.081 mg/mL; TBD, Tianjin, China). Invariant natural killer T cells were sorted using magnetic bead purification following the manufacturer's instruments (Miltenyi Biotec). The purity of the iNKT cells was examined by flow cytometry for FITC‐TCR‐β cells double‐stained with PE‐PBS57/mCD1d tetramer. Invariant natural killer T cells (1.5 × 10^5^/well) were cultured alone or co‐cultured with the indicated numbers of CD4^+^ CD25^−^ T cells from WT mice in round‐bottom 96‐well plates pre‐bound with anti‐CD3 (2 μg/mL) and anti‐CD28 (2 μg/mL) in RPMI 1640 complete medium and restimulated with α‐GalCer (100 ng/mL) in the presence of anti‐IL‐2 mAb (0.2 μg/mL) or IgG isotype mAb. Three days later, cellular components were obtained, and the number of CD4^+^ FoxP3^+^ T cells and the expression of FoxP3 mRNA were measured by flow cytometry and RT‐PCR respectively. The concentration of IL‐10 in supernatants was measured by ELISA following the manufacturer's protocol.

### 2.11 Flow cytometric analysis

The MNCs from lungs were obtained and resuspended in the fluorescence activated cell sorting (FACS) buffer (1‐2 × 10^6^ cells/mL), and then blocked using anti‐CD16/CD32 antibody (clone 2.4G2; BD Biosciences, San Diego, CA) to avoid non‐specific binding and subsequently labelled with isotype controls or the following antibodies: FITC‐CD4, FITC‐IFN‐γ, FITC‐IL‐4 and PeCy7‐FoxP3, PeCy5‐TCR‐β (eBioscience) and PE‐PBS‐57/mCD1d tetramer (gifted by the Natural Institutes of Health tetramer core facility). Invariant natural killer T cells were determined as PBS‐57/mCD1d tetramer and TCR‐β double‐positive cells. For intracellular cytokine staining, lung MNCs were cultured for 4‐6 hours with 50 ng/mL phorbol 12‐myristate 13‐acetate (PMA) and 500 ng/mL ionomycin in the presence of monensin (1 μL/mL) (all from Sigma). Cells were collected and washed, and then intracellular staining was performed following the manufacturer's procedures (eBioscience). Treg cells were measured as CD4^+^ FoxP3^+^ cells. Intracellular staining for FoxP3 was performed using Fix/Perm buffer reagents (eBioscience) according to the manufacturer's protocol. Cells were detected by flow cytometry (BD FACSAria III, BD Biosciences) and the acquired data were analysed using FlowJo software (Tree Star, San Carlos, CA).

### 2.12 Measurement of airway hyperresponsiveness

Mice were anesthetized and inserted with a 20‐gauge polyethylene catheter, and subsequently mechanical ventilation was performed. Dynamic compliance (Cdyn) and airway resistance (RL) to increasing dosages of aerosolized methacholine (Mch), ranging from 3.12 to 50 mg/mL in PBS, for 3 minutes were determined by the FinePointe RC system (Wilmington, NC) to analyse AHR. Non‐specific airway responsiveness was measured through exposing mice to aerosolized PBS to decide the baseline value.

### 2.13 Bronchoalveolar lavage and lung histologic analysis

Mice were euthanized and lungs were lavaged by a catheter with a total volume of 1.5 mL PBS containing 1 mmol/L sodium EDTA, which was followed by lung resection, as previously described.^12^ The levels of IL‐2, IL‐4, IL‐5, IL‐10, IL‐13 and IFN‐γ in the supernatants were assessed by ELISA according to the manufacturer's instructions (eBioscience). Cells recovered from the BALF were collected and stained with May‐Grunwald Giemsa (Jiancheng, Nanjing, China) for differential cell counting.

The right lungs were excised after bronchoalveolar lavage (BAL) and immediately fixed in 4% buffered paraformaldehyde. Subsequently, the samples were dehydrated and embedded in paraffin. Lung tissue sections were stained using haematoxylin‐eosin (HE) staining to evaluate perivascular and peribronchial inflammation and periodic acid‐Schiff (PAS) staining (Baso, Taiwan, China) to assess goblet cell hyperplasia.

### 2.14 Statistical analyses

All data are expressed as the mean ± SD. Statistical analyses were performed with a student's unpaired *t* test or one‐way ANOVA using GraphPad Prism 5 (GraphPad Software Inc., San Diego, CA) software. *P *<* *0.05 was considered statistically significant.

## 3 RESULTS

### 3.1 Intraperitoneal administration of α‐GalCer could promote IL‐10 production through the activation of invariant natural killer T cells in wild‐type mice

**Figure 1:**
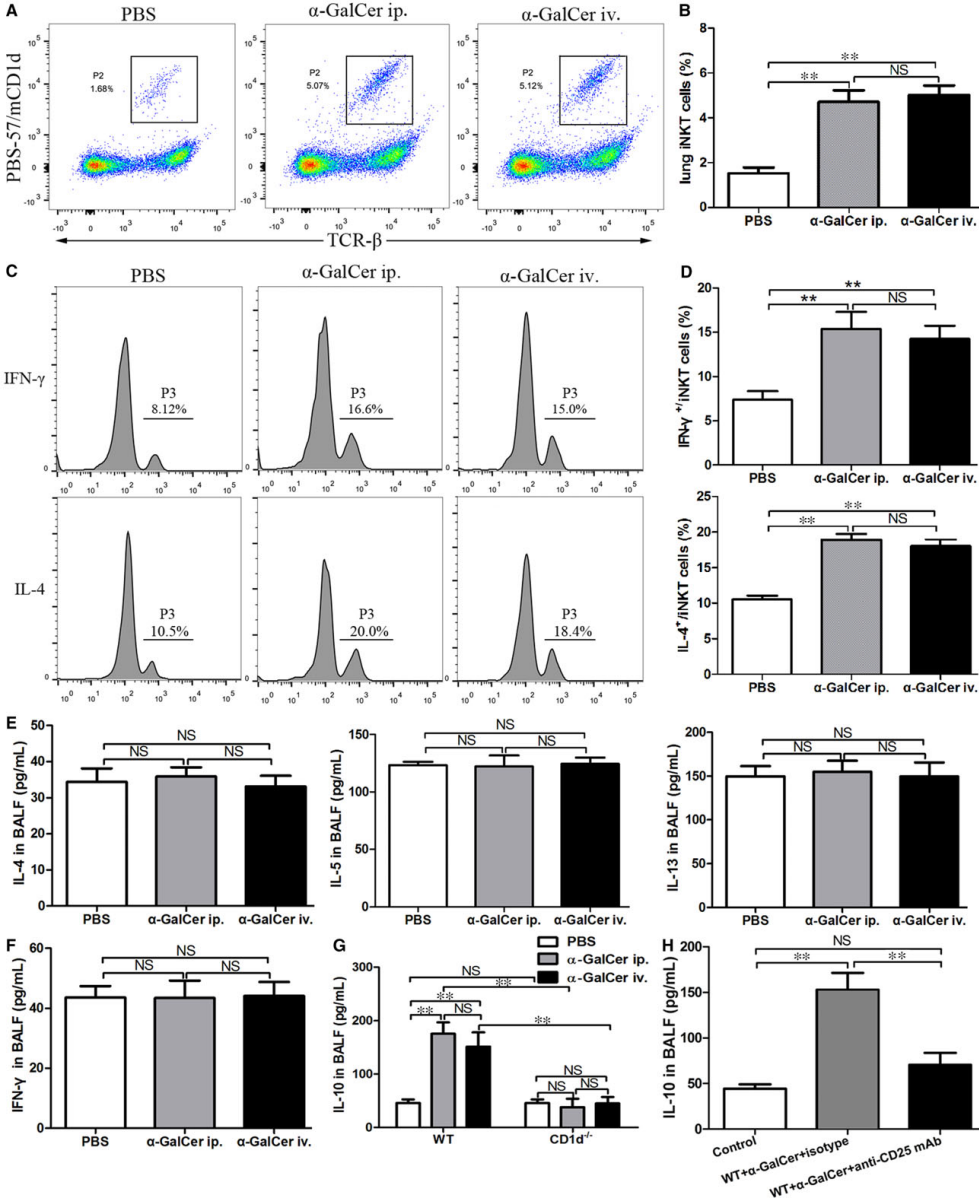
Intraperitoneal administration of α‐galactosylceramide (α‐GalCer) could promote IL‐10 through the activation of iNKT cells in WT mice. A, Lung iNKT cells from WT mice intraperitoneally (ip.) or intravenously (iv.) treated with α‐GalCer or intraperitoneally treated with PBS were determined using flow cytometry. The gating used for lung iNKT cells (PBS‐57/mCD1d^+^ TCR‐β^+^) (gate P2) and the corresponding percentages are indicated in each dot plot. B, Percentages of lung iNKT cells in lung MNCs were determined. C, IFN‐γ (top) and IL‐4 (bottom) production in lung iNKT cells from WT mice intraperitoneally or intravenously treated with α‐GalCer or intraperitoneally treated with PBS were determined. The numbers above the parentheses showed the percentage of positive cells. D, Percentages of IFN‐γ‐ and IL‐4‐producing lung iNKT cells of WT mice intraperitoneally or intravenously treated with α‐GalCer or intraperitoneally treated with PBS. E, The levels of IL‐4, IL‐5 and IL‐13 in the bronchoalveolar lavage fluid (BALF) from WT mice intraperitoneally or intravenously treated with α‐GalCer or intraperitoneally treated with PBS. F, The concentration of IFN‐γ in the BALF from WT mice intraperitoneally or intravenously treated with α‐GalCer or intraperitoneally treated with PBS. G, The concentration of IL‐10 in the BALF from WT mice and CD1d^−/−^ mice intraperitoneally or intravenously treated with α‐GalCer or intraperitoneally treated with PBS. H, The effect of intraperitoneal administration of α‐GalCer on the concentration of IL‐10 in the BALF from WT mice with anti‐CD25 mAb or IgG isotype treatment. Data are shown as mean ± SD of three independent experiments (n = 18) and one representative experiment is indicated. **P <* *0.01. NS, not significant

α‐galactosylceramide, a specific and potent stimulant for iNKT cells, can activate iNKT cells to promote the production of Th1 and Th2 cytokines, such as IL‐4 and IFN‐γ.^5^, ^6^ As shown in Figure 1, intraperitoneal administration of α‐GalCer could enhance the number of lung iNKT cells in WT mice (Figure 1A, B), and also promote the secretion of IL‐4 and IFN‐γ in lung iNKT cells (Figure 1C, D), compared with WT mice treated with PBS (*P *<* *0.01). Next, we sought to evaluate the effect of intraperitoneal administration of α‐GalCer on cytokine production in the BALF from WT mice. As outlined in Figure 1, intraperitoneal administration of α‐GalCer had no significant effect on the concentrations of IL‐4, IL‐5, IL‐13 and IFN‐γ in the BALF, compared with PBS administration (Figure 1E, F) (*P *>* *0.05). Surprisingly, the level of IL‐10 in the BALF was obviously higher in WT mice intraperitoneally treated with α‐GalCer, compared with PBS treatment (Figure 1G) (*P *<* *0.01). Furthermore, our findings showed that no significant difference was found in the number of lung iNKT cells, lung IFN‐γ^+^ iNKT cells and lung IL‐4^+^ iNKT cells, as well as the levels of cytokines (IL‐4, IL‐5, IL‐13, IFN‐γ and IL‐10) in the BALF between WT mice intraperitoneally and intravenously treated with α‐GalCer (Figure 1A‐G) (*P *>* *0.05). Taken together, these results suggested that intraperitoneal administration of α‐GalCer could promote IL‐10 production in the BALF in WT mice.

To determine whether the enhanced concentration of IL‐10 by α‐GalCer in the BALF was dependent on iNKT cells, the level of IL‐10 in the BALF from WT or CD1d‐knockout mice with intraperitoneal administration of α‐GalCer was measured by ELISA. Our data showed that the concentration of IL‐10 in the BALF was markedly higher in WT mice treated with α‐GalCer than that in CD1d‐knockout mice treated with α‐GalCer (Figure 1G) (*P *<* *0.01). However, the level of IL‐10 in the BALF was similar between CD1d‐knockout mice treated with α‐GalCer or PBS (Figure 1G) (*P *>* *0.05). Furthermore, we found that the level of IL‐10 in the BALF was similar in WT and CD1d‐knockout mice intraperitoneally and intravenously treated with α‐GalCer (Figure 1G) (*P *>* *0.05). Thus, these data demonstrated that α‐GalCer‐enhanced IL‐10 production in the BALF was dependent on iNKT cells.

Treg cells can produce IL‐10, a major suppressive cytokine, during the development of immune tolerance.^16^ To evaluate whether IL‐10 is specifically related to Treg cells, the concentration of IL‐10 in the BALF from WT mice treated with anti‐CD25 mAb or IgG isotype mAb 72 hours before intraperitoneal administration of α‐GalCer was measured by ELISA. The concentration of IL‐10 in the BALF from WT mice treated with anti‐CD25 mAb was significantly reduced in comparison to WT mice treated with IgG isotype (Figure 1H) (*P *<* *0.01). Hence, our findings showed that α‐GalCer‐enhanced IL‐10 production in the BALF was probably related to Treg cells in WT mice.

### 3.2 Intraperitoneal administration of α‐GalCer could enhance the expression of FoxP3 mRNA and expansion of CD4^+^ FoxP3^+^ Treg cells in the lung through the activation of iNKT cells

**Figure 2:**
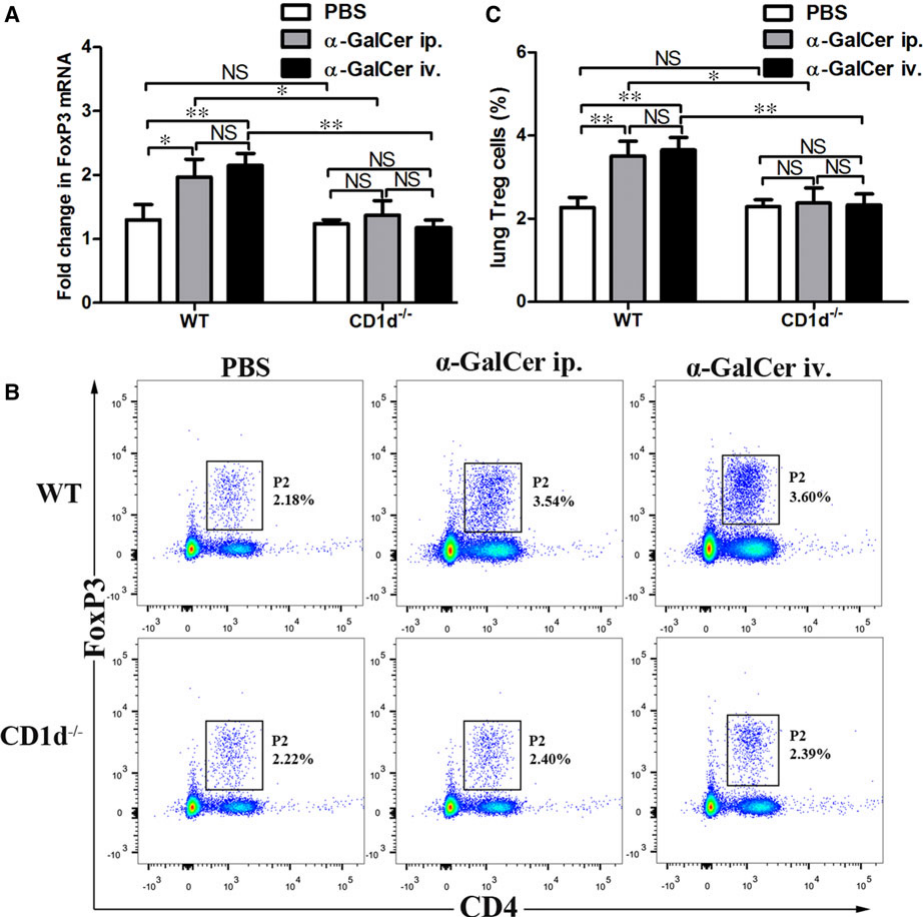
Intraperitoneal administration of α‐GalCer could enhance the expression of FoxP3 mRNA and expansion of CD4^+^ FoxP3^+^ Treg cells in the lung through the activation of iNKT cells in mice. A, Expression of FoxP3 mRNA of lung tissues from WT mice and CD1d‐knockout (CD1d^−/−^) mice intraperitoneally (ip.) or intravenously (iv.) treated with α‐GalCer or intraperitoneally treated with PBS was detected by quantitative RT‐PCR. B, Lung Treg cells were confirmed by CD4 and FoxP3 staining in WT mice or CD1d^−/−^ mice intraperitoneally or intravenously treated with α‐GalCer or intraperitoneally treated with PBS using flow cytometry. The gating used for Treg cells (CD4^+^ FoxP3^+^ Treg cells) (gate P2) and the corresponding percentages are indicated in each dot plot. C, Percentages of lung Treg cells (CD4^+^ FoxP3^+^ Treg cells) in WT mice or CD1d^−/−^ mice intraperitoneally or intravenously treated with α‐GalCer or intraperitoneally treated with PBS. Data are shown as mean ± SD of three independent experiments (n = 18), and one representative experiment is indicated. *P <* *0.05; **P <* *0.01. NS, not significant

CD4^+^ FoxP3^+^ Treg cells represent the principal Treg cell populations and FoxP3 is defined as the master regulator of Treg cells.^17^, ^18^ To assess whether intraperitoneal administration of α‐GalCer may affect the expression of FoxP3 mRNA and percentages of CD4^+^ FoxP3^+^ Treg cells in the lung, the expression of FoxP3 mRNA and number of CD4^+^ FoxP3^+^ Treg cells in the lung from WT mice with intraperitoneal administration of α‐GalCer were examined. As shown in Figure 2, the expression of FoxP3 mRNA in the lung and frequency of lung Treg cells were obviously higher in WT mice intraperitoneally treated with α‐GalCer, compared with PBS treatment (Figure 2A‐C) (*P *<* *0.05 or *P *<* *0.01). Furthermore, our findings showed that no difference was detected in the expression of FoxP3 mRNA in the lung and frequency of lung Treg cells between WT mice intraperitoneally and intravenously treated with α‐GalCer (Figure 2A‐C) (*P *>* *0.05). Thus, intraperitoneal administration of α‐GalCer could enhance the expression of FoxP3 mRNA and expansion of CD4^+^ FoxP3^+^ Treg cells in the lung from WT mice.

To further determine whether iNKT cells are responsible for the elevated expression of FoxP3 mRNA and expansion of CD4^+^ FoxP3^+^ Treg cells induced by α‐GalCer, the expression of FoxP3 mRNA and number of CD4^+^ FoxP3^+^ Treg cells in the lung from CD1d‐knockout mice with intraperitoneal administration of α‐GalCer or PBS were measured. Interestingly, there was no significant difference in the expression of FoxP3 mRNA and number of CD4^+^ FoxP3^+^ Treg cells in the lung between CD1d‐knockout mice treated with α‐GalCer and PBS (Figure 2A‐C) (*P *>* *0.05). In addition, WT mice with α‐GalCer treatment showed a significant increase in the expression of FoxP3 mRNA and number of CD4^+^ FoxP3^+^ Treg cells in the lung, compared with CD1d‐knockout mice treated with α‐GalCer (Figure 2A‐C) (*P *<* *0.05 or *P *<* *0.01). Further, we found that the expression of FoxP3 mRNA in the lung and frequency of lung Treg cells in CD1d‐knockout mice intraperitoneally and intravenously administrated with α‐GalCer were similar (Figure 2A‐C) (*P *>* *0.05). Thus, these data indicated that intraperitoneal administration of α‐GalCer could enhance the expression of FoxP3 mRNA and expansion of CD4^+^ FoxP3^+^ Treg cells in the lung through the activation of iNKT cells in WT mice.

### 3.3 α‐GalCer did not alter IL‐10 production and suppressive activity of CD4^+^ CD25^+^ T cells in vitro

**Figure 3:**
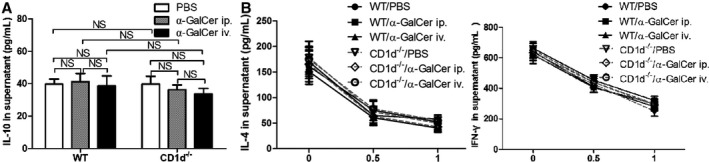
α‐GalCer could not alter IL‐10 production and suppressive activity of Treg cells in vitro. A, Spleen Treg cells from WT mice and CD1d^−/−^ mice treated with intraperitoneal (ip.) or intravenous (iv.) injection of α‐GalCer or intraperitoneal injection of PBS were cultured in vitro for 3 days, and then the concentration of IL‐10 in the supernatants was determined by ELISA. B, CD4^+^ CD25^−^ T cells co‐cultured with the indicated ratio of Treg cells from WT mice or CD1d^−/−^ mice treated with α‐GalCer or PBS for 3 days. The potency of the Treg‐mediated suppression was expressed as the relative inhibition of cytokine (IFN‐γ and IL‐4) production for each Treg/CD4^+^ CD25^−^ T ratio. Data are shown as mean ± SD of three independent experiments (n = 15), and one representative experiment is indicated. NS, not significant

To assess IL‐10 production from CD4^+^ CD25^+^ T cells in WT mice and CD1d‐knockout mice intraperitoneally treated with α‐GalCer or PBS, CD4^+^ CD25^+^ T cells were cultured in vitro for 72 hours, and the supernatants were collected to measure the concentration of IL‐10 by ELISA. We observed that IL‐10 production in the supernatants from WT mice treated with α‐GalCer was not markedly elevated, compared with WT mice treated with PBS (Figure 3A) (*P *>* *0.05), and IL‐10 production in the supernatants was similar between CD1d‐knockout mice treated with α‐GalCer and PBS (Figure 3A) (*P *>* *0.05). Surprisingly, there was no significant difference in the concentration of IL‐10 in the supernatants between WT mice and CD1d‐knockout mice treated with α‐GalCer (Figure 3A) (*P *>* *0.05).

To further evaluate the suppressive capacity of CD4^+^ CD25^+^ T cells, CD4^+^ CD25^−^ T cells co‐cultured with the indicated ratio of CD4^+^ CD25^+^ T cells from WT mice and CD1d‐knockout mice intraperitoneally treated with α‐GalCer or PBS. As outlined in Figure 3, the suppressive influences of Treg cells on cytokine production were gradually enhanced with increasing numbers of Treg cells. Similar to CD4^+^ CD25^+^ T cells from WT mice treated with PBS, CD4^+^ CD25^+^ T cells from CD1d‐knockout mice treated with PBS could inhibit the secretion of IL‐4 and IFN‐γ from CD4^+^ CD25^−^ T cells (Figure 3B) (*P *>* *0.05). Interestingly, the suppressive capacity of CD4^+^ CD25^+^ T cells from WT mice and CD1d‐knockout mice treated with α‐GalCer on production of cytokines was also similar (Figure 3B) (*P *>* *0.05).

Furthermore, our data indicated that no difference was detected in the IL‐10 production and suppressive ability of CD4^+^ CD25^+^ T cells between WT mice with intraperitoneal and intravenous administration of α‐GalCer (Figure 3A, B) (*P *>* *0.05). Additionally, the IL‐10 production and suppressive ability of CD4^+^ CD25^+^ T cells were similar between CD1d‐knockout mice with intraperitoneal and intravenous administration of α‐GalCer (Figure 3A, B) (*P *>* *0.05).

Taken together, Treg cells retained their IL‐10 production and suppressive ability in vitro regardless of the presence or absence of invariant natural killer T cells activated by α‐GalCer.

### 3.4 Intraperitoneal administration of α‐GalCer could induce airway inflammation and airway hyperresponsiveness in wild‐type mice treated with anti‐CD25 mAb

**Figure 4:**
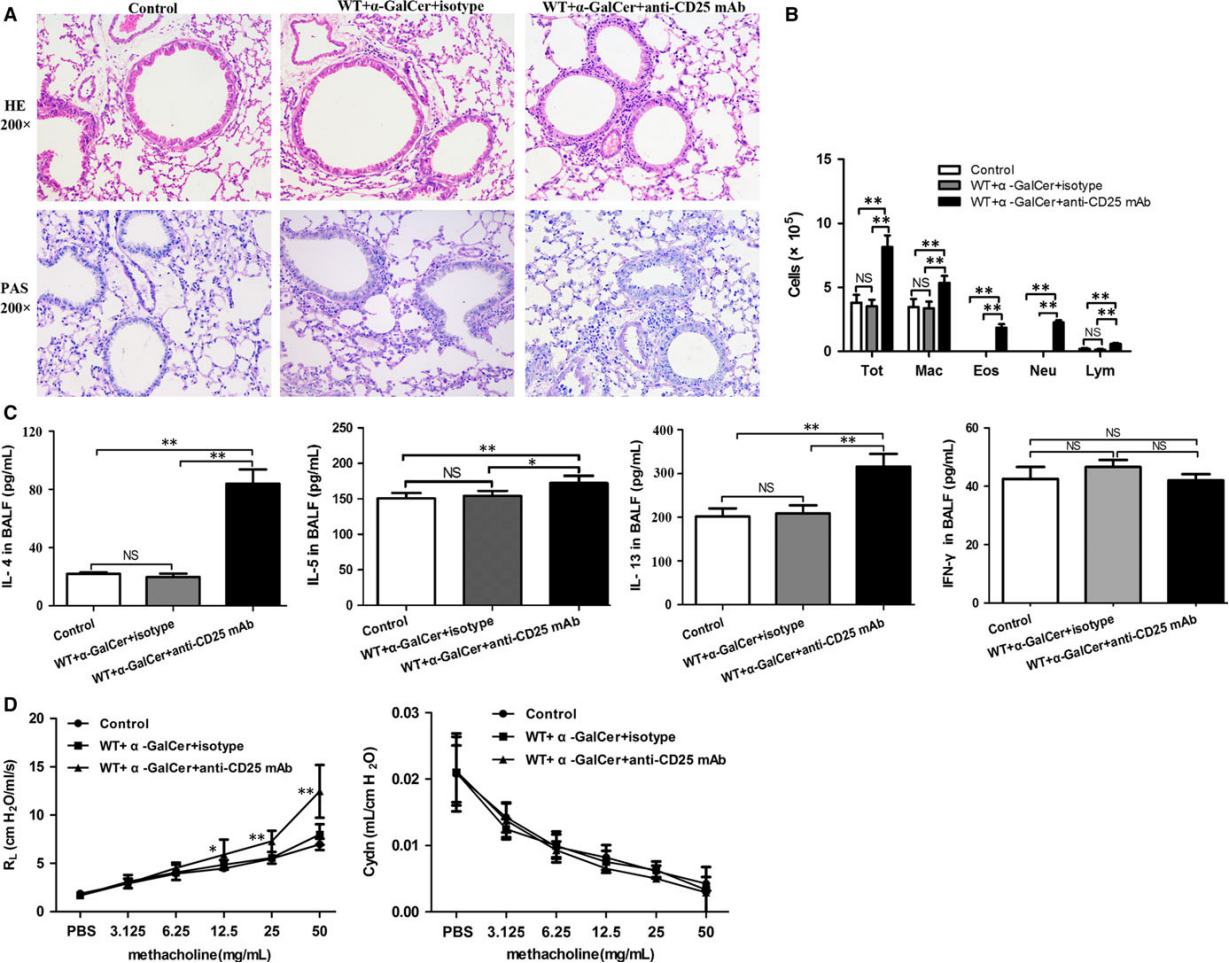
Intraperitoneal administration of α‐GalCer could induce airway inflammation and airway hyperresponsiveness (AHR) in WT mice treated with anti‐CD25 mAb. A, Histopathological analysis of lung tissue from anti‐CD25 mAb‐ or IgG isotype mAb‐treated WT mice intraperitoneally administrated with α‐GalCer or PBS using haematoxylin and eosin (HE) staining and periodic acid‐Schiff (PAS) staining. B, Total cell and differential cell counting in the BALF from anti‐CD25 mAb‐ or IgG isotype mAb‐treated WT mice intraperitoneally administrated with α‐GalCer or PBS. Tot, total cell counts; Eos, eosinophils; Neu, neutrophils; Mar, macrophages; Lym, lymphocytes. C, The concentrations of IL‐4, IL‐5, IL‐13, and IFN‐γ in the BALF from anti‐CD25 mAb‐ or IgG isotype mAb‐treated WT mice intraperitoneally administrated with α‐GalCer or PBS. Data are shown as mean ± SD of three independent experiments (n = 18), and one representative experiment is indicated. *P <* *0.05; **P <* *0.01. NS, not significant. D, Airway response to increasing concentrations of methacholine (Mch) was examined. Data are expressed as mean ± SD of three independent experiments (n = 5), and one representative experiment is indicated. Significant differences between anti‐CD25 mAb‐ or IgG isotype mAb‐treated WT mice intraperitoneally administrated with α‐GalCer or PBS are shown as *P <* *0.05 and **P <* *0.01

Given the findings that anti‐CD25 mAb administration can lead to functional inactivation of Treg cells in vivo,^19^, ^20^ we next investigated whether intraperitoneal administration of α‐GalCer could induce airway inflammation and AHR in WT mice intravenously treated with anti‐CD25 mAb. Our results indicated that intraperitoneal administration of α‐GalCer could result in increased inflammatory cell infiltration in the respiratory tract (Figure 4A), increased number of total cells and eosinophils in the BALF (Figure 4B) (*P *<* *0.01) and elevated levels of IL‐4, IL‐5 and IL‐13 in the BALF (Figure 4C) (*P *<* *0.05 or *P *<* *0.01), but no PAS‐positive goblet cells in the airway epithelium were observed in WT mice treated with anti‐CD25 mAb or with IgG isotype mAb (Figure 4A). However, no difference was found in the levels of IL‐4, IL‐5 and IL‐13 between control group and IgG isotype mAb treatment group (Figure 4C) (*P *>* *0.05). In addition, our findings indicated that intraperitoneal administration of α‐GalCer could induce enhanced RL to Mch in WT mice treated with anti‐CD25 mAb, compared with WT mice treated with IgG isotype mAb (Figure 4D) (*P *<* *0.05 or *P *<* *0.01). However, no difference was observed in terms of Cdyn between the two groups (Figure 4D) (*P *>* *0.05). Collectively, these data showed that intraperitoneal administration of α‐GalCer could induce airway inflammation and AHR in WT mice intravenously treated with anti‐CD25 mAb.

### 3.5 Intraperitoneal administration of α‐GalCer could promote IL‐2 production and expression of IL‐2 mRNA through the activation of invariant natural killer T cells in mice

**Figure 5:**
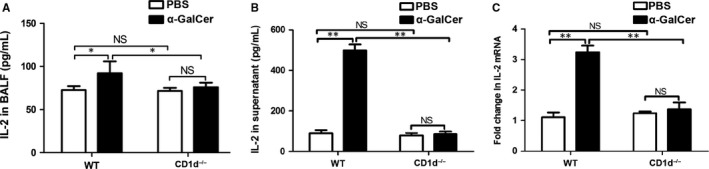
Intraperitoneal administration of α‐GalCer could promote IL‐2 production and expression of IL‐2 mRNA through the activation of iNKT cells in mice. A, BALF were collected from WT mice and CD1d^−/−^ mice intraperitoneally treated with α‐GalCer or PBS, and IL‐2 production was analysed by ELISA. B, Culture supernatants of splenocytes were collected and IL‐2 production was analysed by ELISA. C, Cellular components of culture solution were obtained for RNA extracting and the expression of IL‐2 mRNA was detected by quantitative RT‐PCR. Data are shown as mean ± SD of three independent experiments (n = 18), and one representative experiment is indicated. *P <* *0.05; **P <* *0.01. NS, not significant

IL‐2 is primarily produced by antigen‐activated CD4^+^ T cells, to a lower extent, but also by NKT cells, CD8^+^ T cells, activated dendritic cells and mast cells.^21^, ^22^ To determine whether α‐GalCer could promote IL‐2 production by iNKT cells, BALF and splenocyte from WT or CD1d‐knockout mice were obtained 72 hours after intraperitoneal administration of α‐GalCer or PBS. Our data revealed that intraperitoneal administration of α‐GalCer could enhance the level of IL‐2 in the BALF and splenocyte culture supernatants (Figure 5A, B), as well as the expression of IL‐2 mRNA in culture cellular components (Figure 5C) from WT mice, compared with WT mice treated with PBS (*P *<* *0.05 or *P *<* *0.01). However, no difference was observed in the concentration of IL‐2 in the BALF and splenocyte culture supernatants, as well as the expression of IL‐2 mRNA in culture cellular components between CD1d‐knockout mice intraperitoneally treated with α‐GalCer and with PBS (Figure 5A‐C) (*P *>* *0.05). Furthermore, the level of IL‐2 in the BALF and splenocyte culture supernatants, and expression of IL‐2 mRNA in culture cellular components from WT mice intraperitoneally treated with α‐GalCer were higher than those in CD1d‐knockout mice intraperitoneally treated with α‐GalCer (Figure 5A‐C) (*P *<* *0.05 or *P *<* *0.01). Thus, these results suggested that intraperitoneal administration of α‐GalCer could promote IL‐2 production and expression of IL‐2 mRNA, which requires iNKT cells in WT mice.

### 3.6 Anti‐IL‐2 mAb administration could reduce the number of Treg cells, but did not alter the suppressive function of Treg cells in WT mice intraperitoneally treated with α‐GalCer

**Figure 6:**
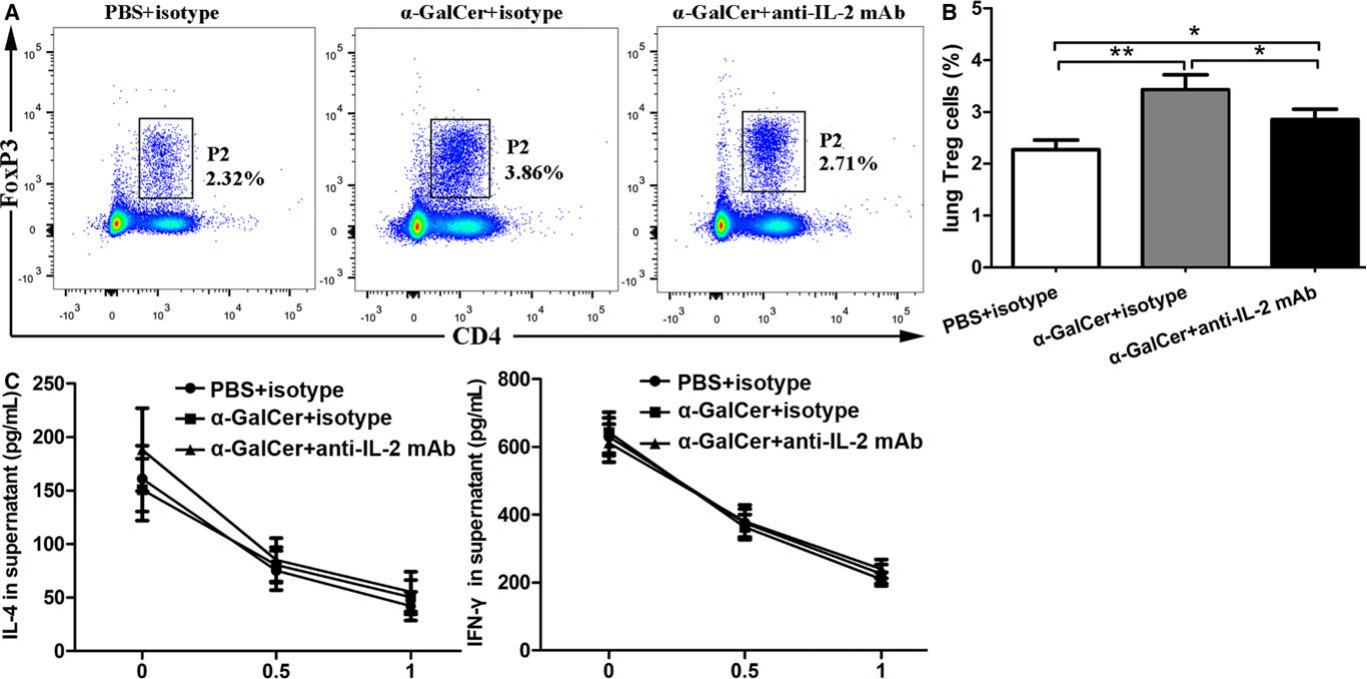
Anti‐IL‐2 mAb administration could reduce the number of Treg cells, but did not alter the suppressive function of Treg cells in WT mice intraperitoneally treated with α‐GalCer. A, Lung Treg cells were confirmed by CD4 and FoxP3 staining in anti‐IL‐2 mAb‐ or IgG isotype mAb‐treated WT mice intraperitoneally administrated with α‐GalCer or PBS using flow cytometry. The gating used for Treg cells (CD4^+^ FoxP3^+^ Treg cells) (gate P2) and the corresponding percentages are shown in each dot plot. B, Percentages of lung Treg cells in anti‐IL‐2 mAb‐ or IgG isotype mAb‐treated WT mice intraperitoneally administrated with α‐GalCer or PBS. C, CD4^+^ CD25^−^ T cells co‐cultured with the indicated ratio of spleen Treg cells isolated from mice for 3 days. The potency of the Treg‐mediated suppression was expressed as the relative inhibition of cytokine (IFN‐γ and IL‐4) production for each Treg/CD4^+^ CD25^−^ T ratio. Data are shown as mean ± SD of three independent experiments (n = 18), and one representative experiment is indicated. *P <* *0.05; **P <* *0.01

The IL‐2 signal plays a major role in driving the development of CD4^+^ FoxP3^+^ Treg cells and is required for the competence of suppressor function and stability of Treg cells.^21^, ^23^, ^24^ Therefore, we sought to explore whether intravenous administration of anti‐IL‐2 mAb could reduce the number and function of Treg cells in WT mice intraperitoneally treated with α‐GalCer. As shown in Figure 6, anti‐IL‐2 mAb administration could significantly reduce the number of lung Treg cells in WT mice intraperitoneally treated with α‐GalCer, compared with IgG isotype mAb administration (Figure 6A, B) (*P *<* *0.05). Interestingly, no difference in the concentrations of IL‐4 and IFN‐γ in the culture supernatants was found between these two groups (Figure 6C) (*P *>* *0.05). Together, these results suggested that anti‐IL‐2 mAb administration could reduce the number of lung Treg cells in WT mice intraperitoneally treated with α‐GalCer, but not change the suppressive function of Treg cells in vitro.

### 3.7 Treg cells induced by invariant natural killer T cells activated by intraperitoneal administration of α‐GalCer involved in IL‐2 in vitro

**Figure 7:**
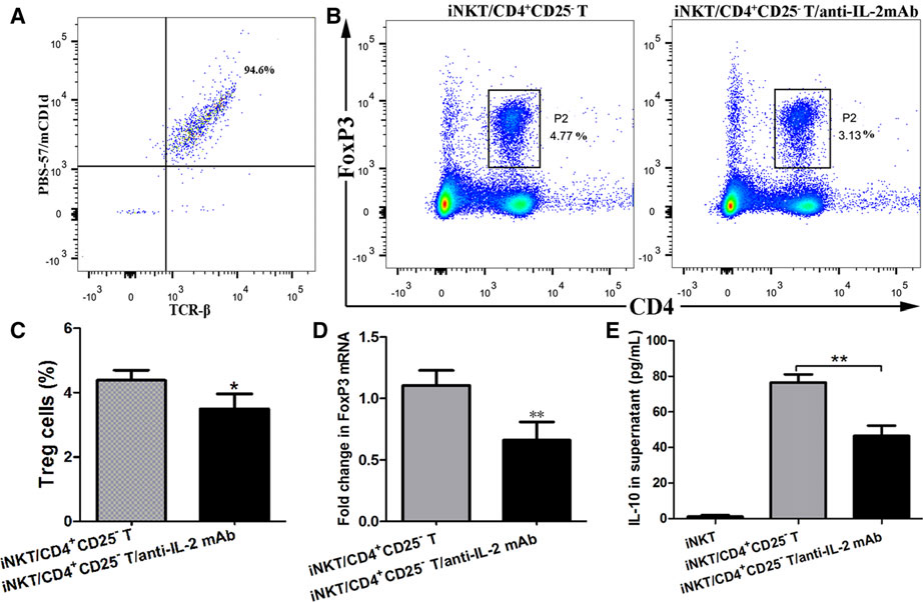
Treg cells induced by α‐GalCer‐activated iNKT cells involved in IL‐2 in vitro. A, Flow cytometry was used to determine the purity of lung iNKT cells, which was stained with both PBS‐57/mCD1d tetramers and a monoclonal antibody against TCR‐β (ie, the proportion of iNKT cells was over 94%). B, Treg cells of cellular components in culture solution were determined by CD4 and FoxP3 staining using flow cytometry. The gating used for Treg cells (CD4^+^ FoxP3^+^ Treg cells) (gate P2) and the corresponding percentages are indicated in each dot plot. C, The percentages of Treg cells from cellular components of culture solution. D, Expression of FoxP3 mRNA of cellular components of culture solution was detected by quantitative RT‐PCR. E, The concentration of IL‐10 in culture supernatants was determined by ELISA. Data are shown as mean ± SD of 3 independent experiments (n = 15), and 1 representative experiment is indicated. *P <* *0.05; **P <* *0.01

To directly investigate the contribution of IL‐2 to the generation of Treg cells induced by α‐GalCer‐activated iNKT cells, we co‐cultured freshly sorted iNKT cells from WT mice intraperitoneally treated with α‐GalCer and CD4^+^ CD25^−^ T cells from naive WT mice in the presence of anti‐IL‐2 mAb or IgG isotype mAb. As indicated in Figure 7A, the purity of iNKT cells, that is, PBS‐57/mCD1d tetramer^+^ TCR‐β^+^ cells, was over 94%. It was found that the anti‐IL‐2 mAb treatment significantly reduced the frequency of CD4^+^ FoxP3^+^ Treg cells in CD4^+^ CD25^−^ T cells (Figure 7B, C) and the expression of FoxP3 mRNA of culture cellular components (Figure 7D), compared with IgG isotype mAb treatment (*P *<* *0.05 or *P *<* *0.01). Furthermore, the level of IL‐10 in the culture supernatants with anti‐IL‐2 mAb treatment was also markedly reduced, compared with IgG isotype mAb treatment (Figure 7E) (*P *<* *0.01). To exclude the possibility that iNKT cells were responsible for the enhanced concentration of IL‐10 in the culture supernatant from the iNKT cells‐CD4^+^ CD25^−^ T cells co‐culture, lung iNKT cells from WT mice intraperitoneally administrated with α‐GalCer were cultured alone. Our result showed that IL‐10 secretion was very limited in the culture supernatant (Figure 7E). Therefore, these results suggested that the generation of Treg cells induced by iNKT cells activated by intraperitoneal administration of α‐GalCer probably involved in IL‐2 in vitro.

### 3.8 ɑ‐GalCer administration had the capacity to inhibit airway inflammation and airway hyperresponsiveness induced by ovalbumin

**Figure 8:**
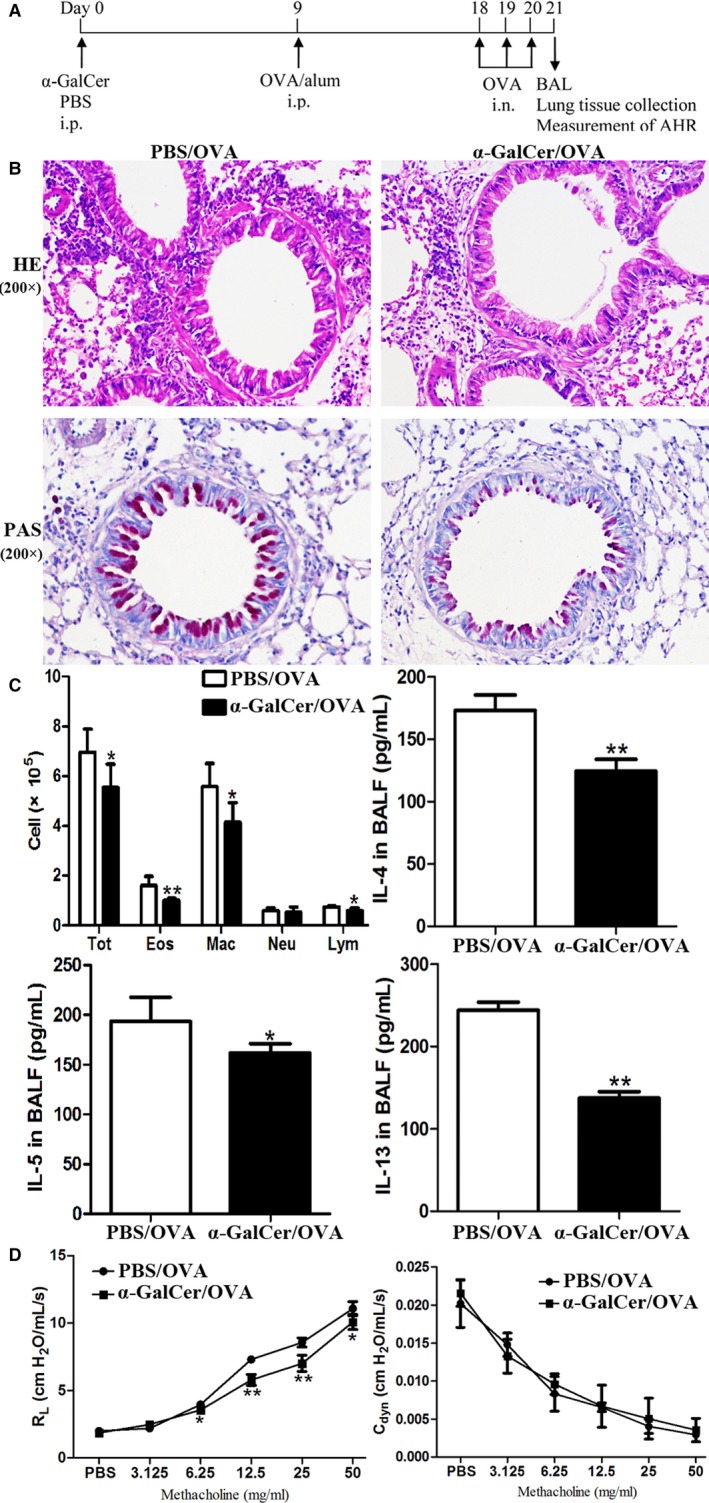
ɑ‐GalCer administration had the capacity to inhibit airway inflammation and AHR induced by ovalbumin (OVA). A, Timeline of the OVA/alum sensitization and OVA challenge protocol and the names of the administration groups (α‐GalCer or PBS). AHR was measured, and then the lung and BALF were obtained 24 h after the final OVA challenge. B, HE and PAS staining of lung sections. C, Total cell, differential cell counting, and the concentrations of cytokines (IL‐4, IL‐5, and IL‐13) in the BALF. Tot, total cell counts; Eos, eosinophils; Neu, neutrophils; Mar, macrophages; Lym, lymphocytes. Data are shown as mean ± SD of 3 independent experiments (n = 18), and 1 representative experiment is indicated. *P <* *0.05; **P <* *0.01. D, Airway response to increasing concentrations of Mch was examined. Data are expressed as mean ± SD of three independent experiments (n = 5), and one representative experiment is indicated. Significant differences are shown as *P <* *0.05 and **P <* *0.01

Finally, we addressed whether intraperitoneal administration of α‐GalCer may induce airway tolerance. The protocol of α‐GalCer administration and OVA immunization/challenge were shown in the Figure 8A. Our findings showed that intraperitoneal administration of α‐GalCer could significantly inhibit the subsequent initiation of airway inflammation as reflected by markedly reduced inflammatory cell infiltration in the airways (Figure 8B), reduced mucus production and PAS‐positive goblet cells in the epithelium of the airways (Figure 8B), and significantly reduced number of eosinophils, macrophages and total cells and strongly decreased concentration of IL‐4, IL‐5 and IL‐13 in the BALF (Figure 8C) (*P *<* *0.05 or *P *<* *0.01). Meanwhile, AHR was significantly reduced, showing that reduced RL to Mch was found in mice intraperitoneally administrated with α‐GalCer, compared with PBS (Figure 8D) (*P *<* *0.05 or *P *<* *0.01). Furthermore, our results revealed that the number of CD4^+^ FoxP3^+^ Treg cells was significantly enhanced in mice treated intraperitoneally with α‐GalCer, compared with PBS (data not shown). Collectively, our data suggested that α‐GalCer administration had the capacity to suppress the induction of airway inflammation and AHR upon following exposure to allergen.

## 4 DISCUSSION

The findings of this study demonstrate that intraperitoneal administration of α‐GalCer can promote IL‐10 production and expansion of CD4^+^ FoxP3^+^ Treg cells in the lung of WT mice, but not CD1d‐knockout mice. However, in vitro culture experiment showed that the activation of iNKT cells do not alter the suppressive activity of CD4^+^ FoxP3^+^ Treg cells. Meanwhile, the functional inactivation of CD4^+^ CD25^+^ Treg cells can induce AHR and airway inflammation in WT mice intraperitoneally treated with α‐GalCer. Furthermore, intraperitoneal administration of α‐GalCer can augment production of IL‐2 in WT mice, but not CD1d‐knockout mice. Moreover, neutralization of IL‐2 can reduce the expansion of lung CD4^+^ FoxP3^+^ Treg cells in vivo and in vitro. Finally, our preliminary findings showed that ɑ‐GalCer administration has the capacity to suppress the induction of airway inflammation and AHR upon following exposure to allergen. Taken together, these results indicate that intraperitoneal administration of α‐GalCer can promote the generation of lung CD4^+^ FoxP3^+^ Treg cells in mice through the release of IL‐2 by the activated iNKT cells.

α‐GalCer, a specific and potent stimulant for iNKT cells, can activate iNKT cells to promote the production of Th1 and Th2 cytokines, such as IL‐4, IL‐13 and IFN‐γ, and thereby modulate a diverse array of immune‐related diseases, including autoimmune disorders, tumour and infection.^5^, ^6^, ^7^ Many studies use mouse models of iNKT cell deficiency to investigate the role of iNKT cells. One model directly targets *J*α*18* (also called *Traj18*), which is needed for iNKT‐TCR formation.^25^ Therefore, *Traj18* ^−/−^ mice are solely devoid of iNKT cells (also called type I NKT cells). However, the diversity of the overall TCR repertoire is impaired in *Traj18* ^−/−^ mice, leading to an almost 60% reduction in the TCRα repertoire diversity.^26^ It is likely that the lower diversity of overall αβ TCR from the original *Traj18* ^−/−^ mice contribute to the divergent results that have been addressed by some of the studies that utilized the mice.^27^ Another model utilizes mice deficient in CD1d, which is necessary for NKT cell development.^28^, ^29^, ^30^ Thus, CD1d deficient (CD1d^−/−^) mice lack both iNKT cells and type II NKT cells. Recently, a new *Traj18* ^−/−^ mice have been prepared by transcription activator‐like effector nuclease (TALEN) methodology.^31^ Zhang et al. 31 showed that TALEN‐*Traj18* ^−/−^ mice (called Jα18 (−10) mice in their paper) lack iNKT cells, and preserve the Jα diversity of the WT animal. However, type Ib NKT cells (also called Vα10 NKT cells) were found in TALEN‐*Traj18* ^−/−^ mice.^31^ Type Ib NKT cells are another rare population of α‐GalCer‐reactive NKT cells, and have the capacity to secrete large amounts of a range of cytokines.^32^ By contrast, type Ib NKT cells were absent from CD1d^−/−^ mice.^31^, ^32^ As such, our present study uses CD1d^−/−^ mice to investigate whether iNKT cells activated by α‐GalCer contribute to the generation of lung Treg cells induced by α‐GalCer. In our previous and present study, intraperitoneal injection of α‐GalCer can induce the activation of lung iNKT cells, but the activation of iNKT cells by α‐GalCer do not result in airway inflammation in WT mice without OVA sensitization and challenge.^12^ As reported by Meyer et al., it seemed that the activation state of iNKT cells may play an important role in the pathogeneses of AHR and airway inflammation, and that α‐GalCer can lead to iNKT cell anergy, which can suppress the subsequent initiation of AHR and airway inflammation in WT mice.^33^ At present, it has been well defined that CD4^+^ CD25^+^ Treg cells can suppress the expansion, activation or function of many other immune cells like Th2 cells and iNKT cells through a mechanism of direct cell‐to‐cell contact and/or such cytokines as IL‐10 and TGF‐β.^17^, ^34^ Therefore, in this study, we investigate whether intraperitoneal administration of α‐GalCer can induce the generation of lung CD4^+^ FoxP3^+^ Treg cells in mice. In our current study, our results showed that intraperitoneal administration of α‐GalCer can promote IL‐10 production and expansion of CD4^+^ FoxP3^+^ Treg cells in the lung of WT mice, but not CD1d‐knockout mice. Therefore, the up‐regulation of IL‐10 production and expansion of CD4^+^ FoxP3^+^ Treg cells induced by α‐GalCer require iNKT cells. Furthermore, functional inactivation of Treg cells in vivo can suppress the production of IL‐10 in the BALF, whereas lung iNKT cells hardly produce IL‐10 in vitro in WT mice intraperitoneally administrated with α‐GalCer. Thus, α‐GalCer‐enhanced IL‐10 production in the lung is probably related to Treg cells in mice. However, in vitro culture experiment showed that iNKT cells activatied by α‐GalCer did not alter the suppressive activities of CD4^+^ FoxP3^+^ Treg cells. Furthermore, the functional inactivation of CD4^+^ CD25^+^ Treg cells with anti‐CD25 mAb can induce AHR and airway inflammation in WT mice intraperitoneally treated with α‐GalCer. Therefore, our findings raise the evidence that intraperitoneal administration of α‐GalCer can drive the expansion of lung CD4^+^ FoxP3^+^ Treg cells through the activated iNKT cells in WT mice. Previous studies have demonstrated that CD4^+^ CD25^+^ Treg cells can suppress the activation of iNKT cells.^35^, ^36^ Therefore, it is probable that lung CD4^+^ FoxP3^+^ Treg cells induced by the activated iNKT cells with intraperitoneal administration of α‐GalCer in turn may be necessary to down‐regulate the activation of iNKT cells and restore them to homeostatic conditions. However, our present results sharply differ from our previous findings showing that intraperitoneal administration of α‐GalCer prior to allergen challenge can promote Th2 response through inducing immunogenic maturation of lung dendritic cells (LDCs) in mouse models of asthma.^12^, ^37^ Therefore, our findings supported the notion that the opposite role of iNKT cells activated by α‐GalCer in different pathological settings may be partially related to the timing of α‐GalCer administration.^38^ Additionally, our present results significantly differ from the previous two reports showing that α‐GalCer administration can abrogate AHR and airway inflammation through the activation of iNKT cells and IFN‐γ production in asthmatic mice.^13^, ^39^

IL‐2 has been suggested to be a T cell growth factor, and play a primary role in the development, survival, and activation of CD4^+^ FoxP3^+^ Treg cells.^23^, ^24^ Previous reports raised some evidence that murine and human NKT cells can express IL‐2 gene and/or IL‐2 protein in the presence or absence of α‐GalCer stimulation.^40^, ^41^ In our present study, our findings revealed that intraperitoneal administration of α‐GalCer can up‐regulate the production of IL‐2 protein and expression of IL‐2 gene in WT mice, but not CD1d‐knockout mice, hence providing the evidence that iNKT cells are responsible for elevated production of IL‐2 in WT mice intraperitoneally administrated with α‐GalCer. Furthermore, although not change the suppressive function of lung CD4^+^ FoxP3^+^ Treg cells, neutralization of IL‐2 can down‐regulate the expansion of lung CD4^+^ FoxP3^+^ Treg cells in WT mice intraperitoneally treated with α‐GalCer. In addition, neutralization of IL‐2 can reduce the secretion of IL‐10, the expression of FoxP3 mRNA and the frequency of CD4^+^ FoxP3^+^ Treg cells in vitro. Thus, our data revealed that it was probable that intraperitoneal administration of α‐GalCer can promote the generation of CD4^+^ FoxP3^+^ Treg cells through the up‐regulation of IL‐2 produced by the activated iNKT cells in WT mice.

In this study, the intraperitoneal injection was used as the route of administration of α‐GalCer, as previously reported,^13^ to investigate that α‐GalCer promoted the generation of lung CD4^+^ FoxP3^+^ Treg cells by activated iNKT cells in WT mice. An important question is that the route of intraperitoneal administration exposes α‐GalCer to the multiple complex immune cells, including peritoneal macrophages and perhaps direct exposure to the liver and this may induce some important biological effects. However, we compared the influence of intraperitoneal administration of α‐GalCer on the expansion and suppressive activity of lung Treg cells with intravenous administration of α‐GalCer in mice. Interestingly, our findings suggested that the effect of the two routes of administration of α‐GalCer on the expansion and suppressive activity of lung Treg cells were similar. Another important question is whether the generation of CD4^+^ FoxP3^+^ Treg cells induced by the activated iNKT cells with intraperitoneal delivery of α‐GalCer requires LDCs. Therefore, thorough investigations are necessary to confirm this in the future.

In conclusion, our study has highlighted that intraperitoneal administration of α‐GalCer can promote the generation of lung CD4^+^ FoxP3^+^ Treg cells in mice through the release of IL‐2 by the activated iNKT cells. It is well known that asthma has fewer and less functional Treg cells, and thus, asthma is a condition of dysregulated immune response.^40^, ^41^ Our preliminary data showed that α‐GalCer administration has the capacity to suppress the induction of airway inflammation and AHR upon following exposure to allergen, suggesting that α‐GalCer administration can induce airway tolerance. Therefore, our present study suggested that it is likely that intraperitoneal administration of α‐GalCer prior to the development of asthma disorder, which can promote the generation of lung CD4^+^ FoxP3^+^ Treg cells by the activated iNKT cells, may have potential as a therapeutic method for asthma. However, an important aspect of prophylactic treatment for allergic diseases is the determination of the time window of allergen sensitization so that the preventive interventions can be performed before allergen sensitization has occurred.^42^ As such, further study in this area will be required.

## CONFLICT OF INTEREST

The authors confirm that there are no conflicts of interest.

## AUTHOR CONTRIBUTIONS

HXN designed and conceived the study; HXN, QHC, LLL, XXG, NSD, ALW, RYL, SC, YH, XHD, HYY and SPH performed the experiments; HXN, QHC, LLL, XXG, ALW, RYL, NSD, YH, XHD, HYY and SPH analysed the data; QHC and SC draw the figures; HXN and QHC wrote and revised the manuscript.

## References

[1] Lantz O , Bendelac A . An invariant T cell receptor alpha chain is used by a unique subset of major histocompatibility complex class I‐specific CD4^+^ and CD4^−^CD8^−^ T cells in mice and humans. J Exp Med. 1994;180:1097‐1106. 7520467 10.1084/jem.180.3.1097 PMC2191643

[2] Rossjohn J , Pellicci DG , Patel O , et al. Recognition of CD1d‐restricted antigens by natural killer T cells. Nat Rev Immunol. 2012;12:845‐857. 23154222 10.1038/nri3328 PMC3740582

[3] Zajonc DM , Cantu C 3rd , Mattner J , et al. Structure and function of a potent agonist for the semi‐invariant natural killer T cell receptor. Nat Immunol. 2005;6:810‐818. 16007091 10.1038/ni1224 PMC2045075

[4] Anderson BL , Teyton L , Bendelac A , Savage PB . Stimulation of natural killer T cells by glycolipids. Molecules. 2013;18:15662‐15688. 24352021 10.3390/molecules181215662 PMC4018217

[5] Bendelac A , Savage PB , Teyton L . The biology of NKT cells. Annu Rev Immunol. 2007;25:297‐336. 17150027 10.1146/annurev.immunol.25.022106.141711

[6] Van Kaer L . Alpha‐galactosylceramide therapy for autoimmune diseases: prospects and obstacles. Nat Rev Immunol. 2005;5:31‐42. 15630427 10.1038/nri1531

[7] Terabe M , Berzofsky JA . The immunoregulatory role of type I and type II NKT cells in cancer and other diseases. Cancer Immunol Immunother. 2014;63:199‐213. 24384834 10.1007/s00262-013-1509-4 PMC4012252

[8] Liu R , La Cava A , Bai XF , et al. Cooperation of invariant NKT cells and CD4^+^ CD25^+^ T regulatory cells in the prevent of autoimmune myasthenia. J Immunol. 2005;175:7898‐7904. 16339525 10.4049/jimmunol.175.12.7898

[9] Ronet C , Darche S , de Moraes ML , et al. NKT cells are critical for the initiation of an inflammatory bowel response against Toxoplasma gondii. J Immunol. 2005;175:899‐908. 16002688 10.4049/jimmunol.175.2.899

[10] Curotto dLM , Lafaille JJ , Graça L . Mechanisms of tolerance and allergic sensitization in the airways and the lungs. Curr Opin Immunol. 2010;22:616‐622. 20884192 10.1016/j.coi.2010.08.014 PMC3900231

[11] Lloyd CM , Hawrylowicz CM . Regulatory T cells in asthma. Immunity. 2009;31:438‐449. 19766086 10.1016/j.immuni.2009.08.007 PMC3385348

[12] Nie H , Yang Q , Zhang G , et al. Invariant NKT cells act as an adjuvant to enhance Th2 inflammatory response in an OVA‐induced mouse model of asthma. PLoS ONE. 2015;10:e0119901. 25830340 10.1371/journal.pone.0119901 PMC4382159

[13] Matsuda H , Suda T , Sato J , et al. α‐galactosylceramide, a ligand of natural killer T cells, inhibits allergic airway inflammation. Am J Respir Cell Mol Biol. 2005;33:22‐31. 15802553 10.1165/rcmb.2004-0010OC

[14] Soroosh P , Doherty TA , Duan W , et al. Lung‐resident tissue macrophages generate Foxp3^+^ regulatory T cells and promote airway tolerance. J Exp Med. 2013;210:775‐788. 23547101 10.1084/jem.20121849 PMC3620360

[15] Sakaguchi S . Naturally arising CD4^+^ regulatory T cells for immunologic self‐tolerance and negative control of immune responses. Annu Rev Immunol. 2004;22:531‐562. 15032588 10.1146/annurev.immunol.21.120601.141122

[16] Palomares O , Martín‐Fontecha M , Lauener R , et al. Regulatory T cells and immune regulation of allergic diseases: roles of IL‐10 and TGF‐β. Genes and Immunty. 2014;15:511‐520. 10.1038/gene.2014.45 25056447

[17] Shevach EM . Mechanisms of Foxp3^+^ T regulatory cell‐mediated suppression. Immunity. 2009;30:636‐645. 19464986 10.1016/j.immuni.2009.04.010

[18] Hori S , Nomura T , Sakaguchi S . Control of regulatory Tcell development by the transcription factor Foxp3. Science. 2003;299:1057‐1061. 28115586

[19] Ly D , Mi QS , Hussain S , Delovitch TL . Protection from type 1 diabetes by invariant NK T cells requires the activity of CD4^+^ CD25^+^ regulatory T cells. J Immunol. 2006;177:3695‐3704. 16951329 10.4049/jimmunol.177.6.3695

[20] Kohm AP , McMahon JS , Podojil JR , et al. Cutting Edge: anti‐CD25 monoclonal antibody injection results in the functional inactivation, not depletion, of CD4^+^ CD25^+^ T regulatory cells. J Immunol. 2006;176:3301‐3305. 16517695 10.4049/jimmunol.176.6.3301

[21] Boyman O , Sprent J . Te role of interleukin‐2 during homeostasis and activation of the immune system. Nat Rev Immunol. 2012;12:180‐190. 22343569 10.1038/nri3156

[22] Powell JD , Ragheb JA , Kitagawa‐Sakakida S , Schwartz RH . Molecular regulation of interleukin‐2 expression by CD28 co‐stimulation and anergy. Immunol Rev. 1998;165:287‐300. 9850868 10.1111/j.1600-065x.1998.tb01246.x

[23] Malek TR , Castro I . Interleukin‐2 receptor signaling: at the interface between tolerance and immunity. Immunity. 2010;33:153‐165. 20732639 10.1016/j.immuni.2010.08.004 PMC2946796

[24] Barron L , Dooms H , Hoyer KK , et al. Cutting edge: mechanisms of IL‐2‐dependent maintenance of functional regulatory T cells. J Immunol. 2010;185:6426‐6430. 21037099 10.4049/jimmunol.0903940 PMC3059533

[25] Cui J , Shin T , Kawano T , et al. Requirement for Valpha14 NKT cells in IL‐12‐mediated rejection of tumors. Science. 1997;278:1623‐1626. 9374462 10.1126/science.278.5343.1623

[26] Bedel R , Matsuda JL , Brigl M , et al. Lower TCR repertoire diversity in Traj18‐deficient mice. Nat Immunol. 2012;13:705‐706. 22814339 10.1038/ni.2347 PMC3748587

[27] Ren Y , Sekine‐Kondo E , Tateyama M , et al. New genetically manipulated mice provide insights into the development and physiologicalfunctions of invariant natural killer T cells. Front Immunol. 2018;9:1294. 29963043 10.3389/fimmu.2018.01294 PMC6010523

[28] Mendiratta SK , Martin WD , Hong S , et al. Mutant mice are deficient in natural T cells that promply produce IL‐4. Immunity. 1997;6:469‐477. 9133426 10.1016/s1074-7613(00)80290-3

[29] Smiley ST , Kaplan MH , Grusby MJ . Immunoglobulin E production in the absence of interleukin‐4‐secreting CD1‐dependent cells. Science. 1997;275:977‐999. 9020080 10.1126/science.275.5302.977

[30] Chen YH , Chiu NM , Mandal M , et al. Impaired NK1^+^ T cell development and early IL‐4 production in CD1‐deficient mice. Immunity. 1997;6:459‐467. 9133425 10.1016/s1074-7613(00)80289-7

[31] Zhang J , Bedel R , Krovi SH , et al. Mutation of the Traj18 gene segment using TALENs to generate natural killer T cell deficient mice. Sci Rep. 2016;6:27375. 27256918 10.1038/srep27375 PMC4891675

[32] Uldrich AP , Patel O , Cameron G , et al. A semi‐invariant Vα10^+^ T cell antigen receptor defines a population of natural killer T cells with distinct glycolipid antigen‐recognition properties. Nat Immunol. 2011;12:616‐623. 21666690 10.1038/ni.2051 PMC5584938

[33] Meyer EH , Goya S , Akbari O , et al. Glycolipid activation of invariant T cell receptor^+^ NK T cells is sufficient to induce airway hyperreactivity independent of conventional CD4^+^ T cells. Proc Natl Acad Sci USA. 2006;103:2782‐2787. 16478801 10.1073/pnas.0510282103 PMC1413796

[34] Thorburn AN , Hansbro PM . Harnessing regulatory T cells to suppress asthma: from potential to therapy. Am J Respir Cell Mol Biol. 2010;43:511‐519. 20097830 10.1165/rcmb.2009-0342TR PMC2970851

[35] Azuma T , Takahashi T , Kunisato A , et al. Human CD4^+^ CD25^+^ regulatory T cells suppress NKT cell functions. Cancer Res. 2003;63:4516‐4520. 12907625

[36] Venken K , Decruy T , Aspeslagh S , et al. Bacterial CD1d‐restricted glycolipids induce IL‐10 production by human regulatory T cells upon cross‐talk with invariant NKT cells. J Immunol. 2013;191:2174‐2183. 23898038 10.4049/jimmunol.1300562

[37] He Q , Liu L , Yang Q , et al. Invariant natural killer T cells promote immunogenic maturation of lung dendritic cells in mouse models of asthma. Am J Physiol Lung Cell Mol Physiol. 2017;313:L973‐L990. 28912381 10.1152/ajplung.00340.2016

[38] Caielli S , Conforti‐Andreoni C , Di Pietro C , et al. On/off TLR signaling decides proinflammatory or tolerogenic dendritic cell maturation upon CD1d‐mediated interaction with invariant NKT cells. J Immunol. 2010;185:7317‐7329. 21078913 10.4049/jimmunol.1000400

[39] Hachem P , Lisbonne M , Michel ML , et al. α‐galactosylceramide‐induced iNKT cells suppress experimental allergic asthma in sensitized mice: role of IFN‐γ. Eur J Immunol. 2005;35:2793‐2802. 16180255 10.1002/eji.200535268

[40] Setoguchi R , Hori S , Takahashi T , Sakaguchi S . Homeostatic maintenance of natural FoxP3^+^ CD4^+^ CD25^+^ regulatory T cells by interleukin (IL)‐2 and induction of autoimmune disease by neutralization. J Exp Med. 2005;201:723‐735. 15753206 10.1084/jem.20041982 PMC2212841

[41] Jiang S , Game DS , Davies D , et al. Activated CD1d‐restricted natural killer T cellsecrete IL‐2: innate help for CD4^+^ CD25^+^ regulatory T cells? Eur J Immunol. 2005;35:1193‐1200. 15770696 10.1002/eji.200425899

[42] Valenta R , Campana R , Marth K , et al. Allergen‐specific immunotherapy: from therapeutic vaccines to prophylactic approaches. J Intern Med. 2012;272:144‐157. 22640224 10.1111/j.1365-2796.2012.02556.x PMC4573524

